# mGluR1/IP3/ERK signaling pathway regulates vestibular compensation in ON UBCs of the cerebellar flocculus

**DOI:** 10.1111/cns.14419

**Published:** 2023-08-25

**Authors:** Dan Liu, Jun Wang, E. Tian, Jingyu Chen, Weijia Kong, Yisheng Lu, Sulin Zhang

**Affiliations:** ^1^ Department of Otorhinolaryngology, Union Hospital, Tongji Medical College Huazhong University of Science and Technology Wuhan China; ^2^ Institute of Otorhinolaryngology, Union Hospital, Tongji Medical College Huazhong University of Science and Technology Wuhan China; ^3^ Department of Physiology, School of Basic Medicine Huazhong University of Science and Technology Wuhan China; ^4^ Institute of Brain Research, Collaborative Innovation Center for Brain Science Huazhong University of Science and Technology Wuhan China

**Keywords:** mGluR1α, unipolar brush cells, vestibular compensation

## Abstract

**Aims:**

To investigate the role of mGluR1α in cerebellar unipolar brush cells (UBC) in mediating vestibular compensation (VC), using mGluR1α agonist and antagonist to modulate ON UBC neurons, and explore the mGluR1/IP3/extracellular signal‐regulated kinase (ERK) signaling pathway.

**Methods:**

First, AAV virus that knockdown ON UBC (mGluR1α) were injected into cerebellar UBC by stereotactic, and verified by immunofluorescence and western blot. The effect on VC was evaluated after unilateral labyrinthectomy (UL). Second, saline, (RS)‐3,5‐dihydroxyphenylglycine (DHPG), and LY367385 were injected into tubes implanted in rats at different time points after UL separately. The effect on ON UBC neuron activity was evaluated by immunofluorescence. Then, Phosphoinositide (PI) and p‐ERK1/2 levels of mGluR1α were analyzed by ELISA after UL. The protein levels of p‐ERK and total ERK were verified by western blot. In addition, the effect of mGluR1α activation or inhibition on VC‐related behavior was observed.

**Results:**

mGluR1α knockdown induced VC phenotypes. DHPG increased ON UBC activity, while LY367385 reduced ON UBC activity. DHPG group showed an increase in PI and p‐ERK1/2 levels, while LY367385 group showed a decrease in PI and p‐ERK1/2 levels in cerebellar UBC of rats. The western blot results of p‐ERK and total ERK confirm and support the observations. DHPG alleviated VC‐related behavior phenotypes, while LY367385 exacerbated vestibular decompensation‐like behavior induced by UL.

**Conclusion:**

mGluR1α activity in cerebellar ON UBC is crucial for mediating VC through the mGluR1/IP3/ERK signaling pathway, which affects ON UBC neuron activity and contributes to the pathogenesis of VC.

## Graphical abstract

Schematic diagram of mGluR1/IP3/ERK signaling pathway in cerebellar ON UBC. Mossy fibers transmit signals to UBCs, which relay them to Purkinje cells via parallel fibers. ON UBCs, regulated by mGluR1α receptors, exhibit prolonged depolarization. mGluR1α activation triggers intracellular signaling (G proteins, PKC, IP3, ERK) and mediates vestibular compensation via the mGluR1/IP3/ERK pathway.

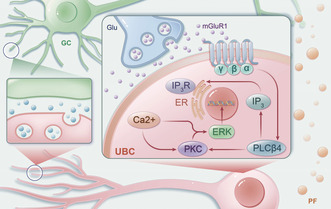

## 1 INTRODUCTION

Vestibular compensation (VC) is a process of adaptation and recovery that occurs in central nerve system following unilateral peripheral vestibular damage.^1^, ^2^, ^3^ The vestibular system principally targets the medial vestibular nuclei (MVN) and the cerebellum, with the MVN serving as the primary processor of vestibular inputs. Upon unilateral vestibular damage, the spontaneous activity in MVN neurons was re‐balanced and the process can modulate VC, and this rebalancing process is regulated by the cerebellum. The flocculus, a major lobe of the cerebellum, projects to the MVN and plays a critical role in the vestibulo‐ocular reflex. The cerebellum receives inputs from various structures, including the MVN and spinal cord, via mossy fibers, which form synapses with granule cells and interneurons in the granular layer. Granule cells provide feedforward control to Purkinje cells and are feedforward controlled by interneurons, including unipolar brush cells (UBCs) and Golgi cells. UBCs contribute to feedforward excitation, while Golgi cells are responsible for inhibitory control. Thus, the proper functioning of UBCs and Golgi cells is crucial for the normal operation of the flocculus.

UBCs in the flocculus fall into two categories, that is, ON and OFF subtypes, in terms of their receptor expression in response to input signals from mossy fibers. OFF UBCs exhibit a suppressed firing activity mediated by the inhibitory receptor type II metabotropic glutamate receptors (mGluR2/3).^4^, ^5^ On the other hand, ON UBCs display activated firing through the excitatory receptor mGluR1α.^6^, ^7^ The diametrically opposite expression patterns of the two subtypes complement each other in the regulation of VC.^8^ Moreover, Gliddon et al.^9^ demonstrated that administration of mGluR1 antagonists effectively mitigated spontaneous nystagmus in the MVN after unilateral vestibular damage. However, the how they are involved in the recovery following peripheral vestibular damage remain unknown.

mGluR1 initiates the downstream signal transduction pathways, the signaling molecules including protein kinase C (PKC), inositol 1,4,5‐triphosphate (IP3), and diacylglycerol.^10^, ^11^ This activation subsequently activates the extracellular signal‐regulated kinase (ERK) signaling pathway. ERK plays a crucial role in the regulation of cellular responses by transmitting external stimuli from the cytoplasm to the nucleus, thereby modulating substrate protein or expression of some genes, including immediate early genes. On the basis of the aforementioned findings,^8^ we are led to postulate, for the first time, that the mGluR1/IP3/ERK signaling pathway in ON UBCs within the cerebellar flocculus may well play a pivotal role in the post‐unilateral labyrinthectomy (UL) mediation of VC.

To verify our hypothesis, we created a knockdown model and used pharmacological interventions to examine the role of mGluR1α in VC. We then evaluated the effect of these interventions on cerebellar neurons and downstream signal transduction pathways associated with VC. This study focused on the critical involvement of mGluR1α in cerebellar UBCs in the mediation of VC via the mGluR1/IP3/ERK signaling pathway.

## 2 MATERIALS AND METHODS

### 2.1 Animals

Male Sprague Dawley (SD) rats aged 8~10 weeks and weighted 180–200 g were obtained from the Experimental Animal Research Center of Hubei Province and properly treated by following the Guidelines for the Care and Use of Laboratory Animals.^12^ Animals were group‐housed at a constant temperature of 22 ± 1°C and in 65 ± 5% humidity under a 12‐h light/dark cycle, with an ad libitum access to food and water. The study was approved by the Animal Ethics Committee of Huazhong University of Science and Technology, Wuhan, China (Serial No: 82071508).

### 2.2 Unilateral labyrinthectomy

UL^13^, ^14^ was performed on the right side of male SD rats aged 8–10 weeks under anesthesia. The operator made an incision, opened the tympanic bulla wall, removed the tympanic bulla, malleus, and incus, and destroyed the vestibule with absolute alcohol. A sham operation was done on the control rats without destroying the tympanic membrane and ossicles.

### 2.3 Exclusion criteria

Animals were excluded from the study if any of the following symptoms appeared^15^: (1) a body weight loss of more than 20%, (2) corneal ulceration, which might result from an inadvertent facial nerve damage, (3) tympanic cavity bleeding, and (4) abnormal behaviors, such as convulsions, paresis or hemiataxia.

### 2.4 Behavioral assessment

Blinded assessors scored the static symptoms of vestibular imbalance,^8^, ^13^, ^14^, ^16^, ^17^ including spontaneous nystagmus, head tilt, postural asymmetry, and tall‐hanging test, presented in behavioral tests. Spontaneous nystagmus was recorded by a video eye‐tracking system and scored on a scale of 6~10 points in terms of bpm. If the nystagmus vanished, air‐blowing over the head was used to evoke it. The result was scored on a six‐point scale. Head tilt was graded in terms of the angle between the jaw and the horizontal plane, with 90° angle listed as 10 points, 60° angle as seven points, and 45° angle as five points, when the animal was made to lie or barrel‐roll on the side of the lesion. Postural asymmetry was rated as follows: spontaneous barrel rolling was awarded 10 points; barrel rolling elicited by a light touch or a puff of air nine points; the recumbent position on the deafferented side without leg support eight points; need of ipsilesional leg support 7–6 points; movement with bilateral leg support five points; occasional postural asymmetry 4–3 points; barely perceptible postural asymmetry 2–1 point(s). Tall‐hanging test was conducted by hoisting animals off the ground by their tails. Results were graded on a 0–10 point scale, with angles between 0° and 180° listed as 0–6 points, and angles between 180° and 360° or over as 8–10 points.

### 2.5 Stereotaxic virus injection

(1) Anesthesia: Male SD rats were anesthetized with pentobarbital sodium (40 mg/kg, ip) and fixed on a dual‐arm stereotaxic instrument with ear rods at a height of 10 mm on both sides and a fixed rod at 12 mm on the head. The head hair was shaved off, and tinfoil was used to shield the eyes from cold light. Indoor temperature was controlled to keep the rats warm throughout the experiment. (2) Leveling: After fixation of the rats, the scalp was cut open and disinfected. The fascial tissue was removed to expose the skull, and the anterior and posterior fontanelles were observed. The head was adjusted to put fontanelles at right levels, front to back and left to right, with the height difference being less than or equal to 0.06 mm. (3) Borehole: After leveling, the distance between the anterior and posterior fontanelles was recorded and converted to the actual coordinates of the target brain area (AP = −4.5 mm, ML = −10 mm, DV = 7.8 mm). The skull surface was marked at/in the target brain area on the basis of the coordinates and then drilled to make a hole. Care should be exercised not to damage the rat's brain by applying too much force while drilling. (4) Virus injection: To inject the virus, a 10 μL glass microsyringe was used, and 400 nL of virus (NC group: pAAV‐U6‐shRNA(NC)‐CMV‐EGFP‐WPRE and mGluR1α‐shRNA group: pAAV‐U6‐shRNA(Grml)‐CMV‐EGFP‐WPRE) was injected into the target brain area at a speed of 50 nL/min. After the injection, the needle was allowed to stay for 15 min to prevent virus leakage and backflow. (5) Postoperative treatment: After surgery, the wound was disinfected and the scalp was closed by suturing. The rat was taken off the stereotaxic apparatus, placed on an electric blanket to maintain body temperature, and returned to a cage with access to food and water.

### 2.6 Immunostaining

Rats were anesthetized and perfused with saline and PFA solution. The cerebellum was dissected, post‐fixed and OCT‐embedded for cryostat sectioning. Sections of 20 μm thickness were prepared, blocked with PBS containing 10% normal donkey serum and 0.1% Triton X‐100, and then incubated with primary antibodies against c‐fos (1:200; Immunoway, USA, Code No: YM3469) and mGluR1α (1:200; BD Pharmingen, Code No: 556389) overnight at 4°C. After washing with PBS, sections were incubated with fluorescent‐labeled secondary antibodies (mixture of Alexa Fluor 488, Donkey Anti‐mouse, 1:200, Jackson ImmunoResearch, Code No: 715‐545‐151) and Alexa Fluor 680, Donkey Anti‐Rabbit, 1:200, Jackson ImmunoResearch, Code No: 711‐625‐152) for 1 h and mounted on glass slides. Images were acquired by using a Zeiss microscope LSM 800 System and analyzed for neuron counts using Image‐Pro‐Plus software. All images were obtained under the identical conditions, and histologically quantified performed by a blinded investigator.

### 2.7 Western Blot

Flocculus tissue was isolated on ice and homogenized in an ice‐cold RIPA Lysis Buffer (Beyotime) with proteinase inhibitors. The supernatant was collected by centrifugation at 12,000 rpm for 15 min at 4°C. Protein concentration was determined by using an Enhanced BCA Protein Assay Kit (Beyotime). Protein lysates (20 μg) were separated on 12% SDS‐polyacrylamide gels and transferred to PVDF membranes. The membranes were blocked and incubated with primary antibodies overnight at 4°C. The primary antibodies used were mouse‐anti‐mGluR1α (1:1000, BD Pharmingen, Code No: 556389), rabbit‐ERK (1:1000, Immunoway, Code No: YT1625), rabbit‐p‐ERK (1:1000, Immunoway, Code No: YP0101), rabbit‐α‐Tubulin (1:4000, Abclonal, Code No: AC003), and rabbit‐anti‐β‐actin (1:5000, Antgene, Code No: ANT321). The membranes were then incubated with HRP‐conjugated secondary antibodies (1:3000, AntGene) and washed to remove excess primary antibody. Bands were detected by employing BeyoECL Plus (Beyotime) and digitally quantified by utilizing Image‐Pro Plus 6.0 software (Media Cybernetics, Inc.).

### 2.8 Implantation of catheter and administration of drugs through the catheter

(1) Anesthesia: The same method was used with stereotaxic virus injection. (2) Leveling: The same technique was employed with stereotaxic virus injection. (3) Borehole: The hole‐boring was the same as that used in stereotaxic virus injection. (4) Burial of skull screws: In addition to the target brain area, a mini screwdriver was utilized to drive skull screws into the drilled holes. (5) Cannula insertion: A cannula of the specified parameters (C = 9 mm, G2 = 0, G1 = 0.5 mm) was introduced by using a cannula holder. The cannula was adjusted to zero position with micromanipulation, and then inserted to the target brain area against the stereotactic coordinates obtained. The cannula was advanced downwards to the target brain area. (6) Dental cement fixation: Mental cement powder and liquid were mixed, with the mixture applied to the skull screw and cannula site, and allowed to bind tightly to the rat skull. (7) Postoperative recovery/treatment: Postoperative treatment was the same as that for stereotaxic virus injection. (8) Cannula administration: The rats were medicated via the injection cannula at a dose of 1000 nL at a rate of 100 nL/min, and the needle was allowed to stay for 5 min after the procedure ended. The rat was put back to its feeding cage after the procedures.

### 2.9 ELISA

Sample collection and processing: Flocculus tissue was homogenized with PBS and centrifuged, with the supernatant collected for testing. Standard wells were prepared with different concentrations of standards. Test samples were diluted with sample diluent. The enzyme marker was added and the resultant sample was incubated at 37°C for 30 min. The samples were then washed with diluted washing solution and then color developers A and B were added. The samples were then incubated at 37°C for 10 min in the dark. Finally, stop solution was added and the OD values were measured at 450 nm. A standard curve was plotted and the actual concentration of the sample was calculated.

### 2.10 Statistical analysis

All data were expressed as Mean ± standard error of the mean (SEM). GraphPad prism 9.0 for analysis statistics. Prior to analysis, the normal distribution test was performed using the Shapiro–Wilk test. When the data followed a normal distribution, the two‐tailed Student's *T*‐test, one‐way ANOVA followed by Tukey's post hoc test, or two‐way ANOVA with Bonferroni post hoc test were employed. In cases where the data did not conform to a normal distribution, the Mann–Whitney test was utilized. A *p* < 0.05 was considered to be statistically significant.

## 3 RESULTS

### 3.1 mGluR1α knockdown in flocculus

**FIGURE 1:**
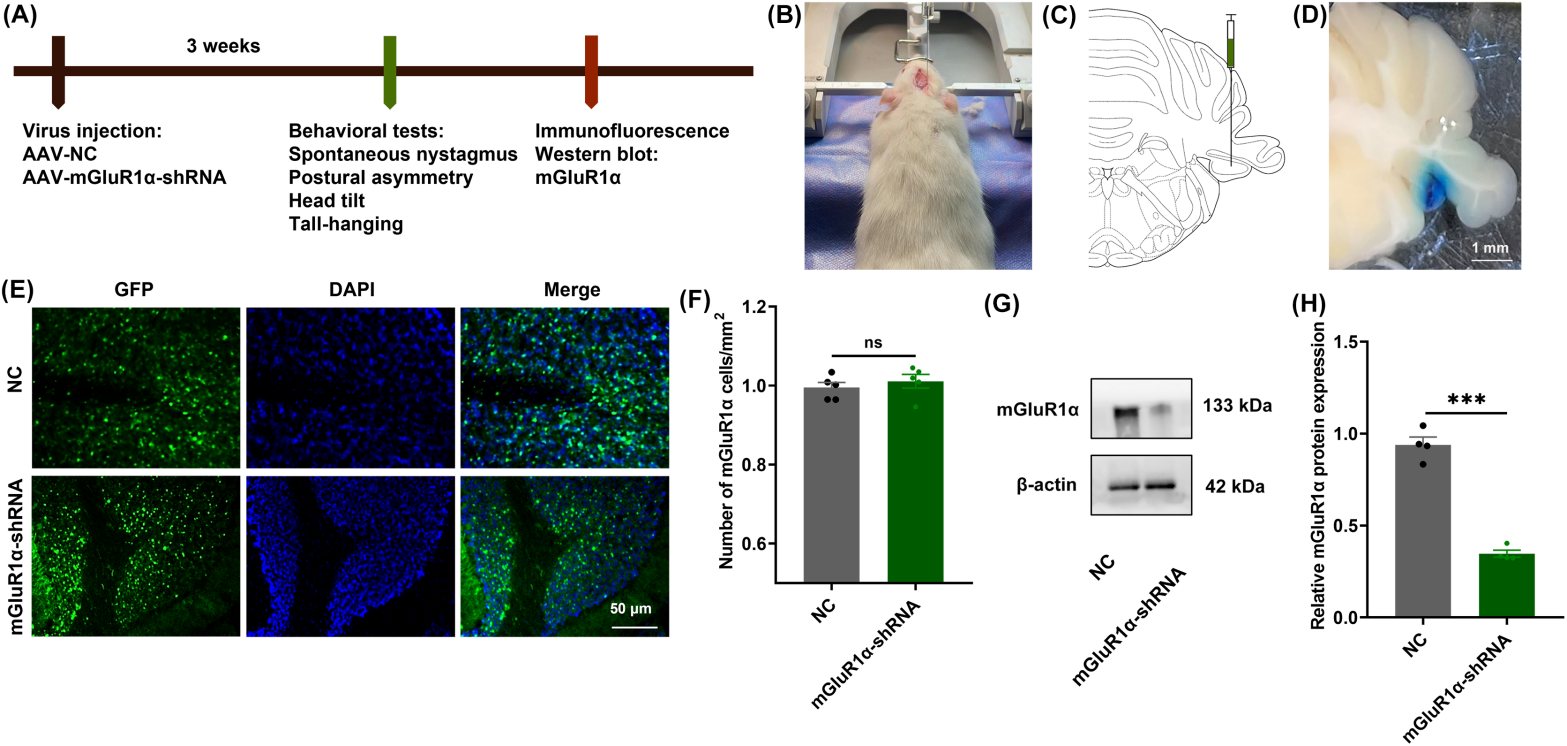
mGluR1α was knockdown in ipsilateral flocculus of UL. (A) Experimental design. AAV‐NC or AAV‐mGluR1α‐shRNA were stereotaxically injected into the ipsilateral cerebellar flocculus of UL. After 3 weeks, confirmed knockdown by immunofluorescence and western blot. Conducted behavioral tests to analyze phenotypes: spontaneous nystagmus, postural asymmetry, head tilt, and tail suspension. (B, C) Stereotactic injection schematic (relative to bragma coordinates: AP = −4.5 mm, ML = −10 mm, DV = 7.8 mm). (D) Cerebellar flocculus injection coordinates were confirmed by methylene blue. (E, F) Fluorescence images of GFP in flocculus after virus infection. The number of GFP‐positive cells were similar in both groups. (G, H) The expression of mGluR1α was significantly reduced in the flocculus after AAV‐mGluR1α‐shRNA injection, confirmed by western blot. UL, unilateral labyrinthectomy; NC, normal control. ns, indicates no statistical difference; ***p < 0.001.

To investigate whether mGluR1α in UBC is involved in VC, we specifically knocked down mGluR1α by stereotaxically injecting AAV‐ mGluR1α‐shRNA into FL (Figure 1A–D) and into ipsilateral flocculus of UL. The injection site was verified by using methylene blue (AP = −4.5 mm, ML = −10 mm, DV = 7.8 mm; Figure 1C,D). Three weeks after AAV injection, the expression of AAV‐ mGluR1α‐shRNA and the AAV‐NC expression was confirmed by their GFP in FL (Figure 1E,F). The western blot results showed that mGluR1α expression was significantly reduced in FL (*t* = 7.703, *p* = 0.0003; Figure 1G,H).

### 3.2 The behavioral phenotypes of VC upon induced by down‐regulated mGluR1α expression in the cerebellar flocculus

**FIGURE 2:**
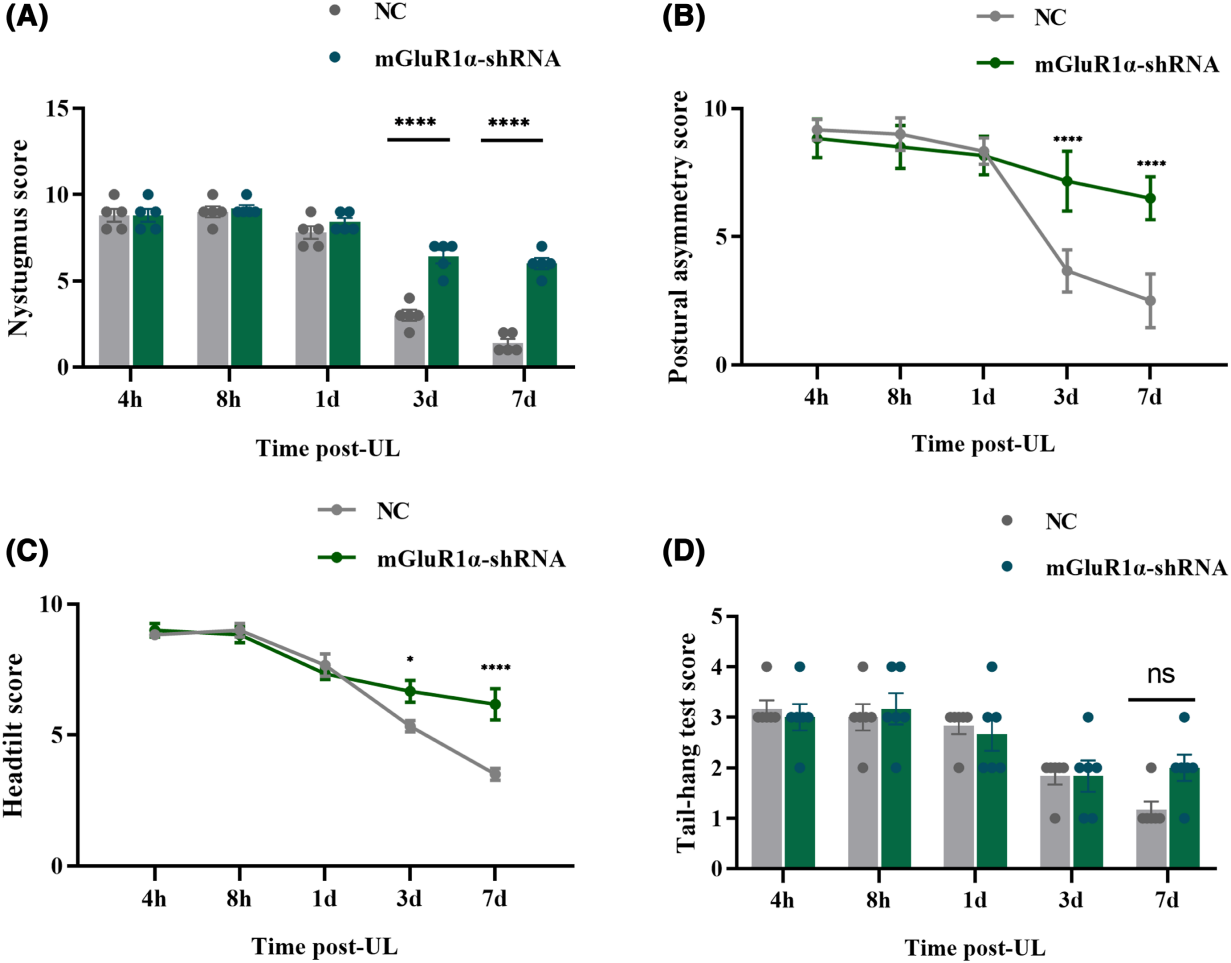
mGluR1α downregulation in ipsilateral cerebellar flocculus impairs VC 3 days after UL. Downregulation of mGluR1α expression affected spontaneous nystagmus (A), postural asymmetry (B), head tilt (C), and tail‐hang test (D), compared to the NC group. Downregulation of mGluR1α expression showed no significant difference in the tail‐hanging test compared to the NC group. UL, unilateral labyrinthectomy; NC, normal control; VC, vestibular compensation. ns, indicates no statistical difference; *p < 0.05; ***p < 0.001; ****p < 0.0001.

Three weeks after knockdown of mGluR1α in ipsilateral flocculus, tests on spontaneous nystagmus, postural asymmetry, head tilt, and the tail‐hanging test were conducted to evaluate VC (Figure 2A–D). In all these behavior tests, animals in both groups exhibited an incremental recovery from UL over time. No significant difference was found in the tail‐hanging test between the two groups (Figure 2D; Two‐way ANOVA, *F*(4,50) = 1.409, *p* = 2.446). However, SN (Two‐way ANOVA, *F*(4,40) = 21.19, *p* < 0.0001), postural asymmetry (Two‐way ANOVA, *F*(4,40) = 23.29, *p* < 0.0001), and head tilt (Two‐way ANOVA, *F*(4, 50) = 21.19, *p* = 0.0001) recovered much more slowly in mGluR1α downregulation group than in the control group. Interestingly, post‐hoc analysis revealed no difference among the results of these three tests 1 day before the UL, and only 3 days after UL, was the VC recovery in mGluR1α downregulation group was significantly slowed down, suggesting the mGluR1α plays an important role in ON UBCs of the flocculus.

### 3.3 mGluR1α agonist DHPG activates while antagonist LY367385 inhibits ON UBCs in the flocculus

**FIGURE 3:**
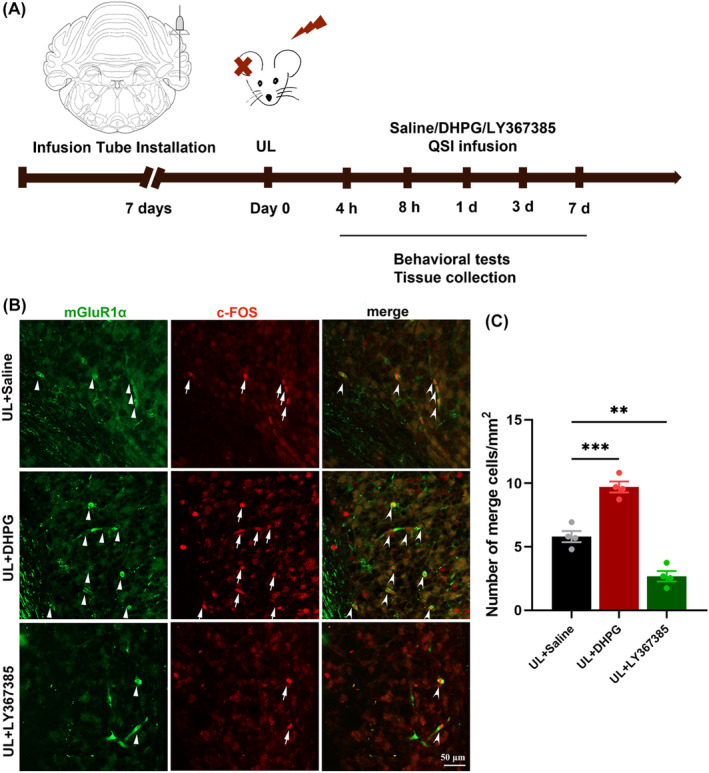
Immunofluorescence staining investigated the effects of drug intervention on the activity of ON UBC neurons. (A) Experimental design of pharmacological interventions. Rats were implanted with catheters and injected with saline, DHPG, or LY367385 at different time points after undergoing UL. Behavioral assessments were conducted daily and tissues were collected after drug administration. (B) Immunofluorescence staining on the activity of ON UBC neurons. LY367385 decreased co‐localized positive neurons for mGluR1α and c‐fos, while DHPG increased them, as shown by immunofluorescence staining in the cerebellar flocculus of rats. (C) Quantitative analysis of the number of co‐localized positive neurons for mGluR1α and c‐fos. UL, unilateral labyrinthectomy; UBC, unipolar brush cells; DHPG, mGluR1α agonist; LY367385, mGluR1α antagonist. **p < 0.01; ***p < 0.001. Scale bar = 50 μm.

To investigate whether acute activation and inhibition mGluR1α could, respectively, promote and impede VC, mGluR1α agonist DHPG and antagonist LY367385 were injected into the ipsilateral flocculus through a cannula, 4, 8 h, 1, 3, and 7 days after UL (Figure 3A). The rats were perfused half an hour after the last dose. DHPG increased while LY367385 decreased the activity of ON UBC neurons, as revealed by the number of co‐localized neurons positive for mGluR1α and c‐fos (Figure 3B,C).

### 3.4 Activation and inhibition of mGluR1α respectively increases and decreases VC

**FIGURE 4:**
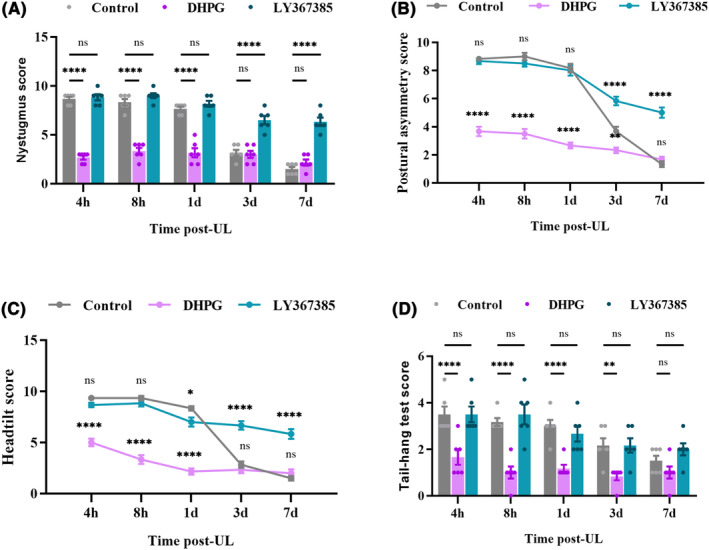
Effects of mGluR1α agonists and antagonists on vestibular compensation behavior. (A) SN of rats was recorded by electrooculography. DHPG reduced SN in rats after UL, with significant effects observed at 4, 8 h, and 1 day. In contrast, LY367385 increased SN in rats at 3 and 7 days compared to the Saline group. (B) Posture asymmetry of rats was assessed. DHPG reduced posture asymmetry in rats at 4, 8 h, 1, and 3 days compared to Saline group, while LY367385 increased posture asymmetry in rats at 3 and 7 days compared to Saline group. (C) The head tilt angle of rats was evaluated. The DHPG group suppressed UL‐induced SN in rats compared to the Saline group, with significant results at 4, 8 h, and 1 day. In contrast, the LY367385 group increased the head tilt of rats at 1, 3, and 7 days compared to the Saline group. (D) The tail hanging test score of rats was assessed. DHPG significantly reduced the tail hanging test score compared to Saline at 4, 8 h, 1, and 3 days, while no significant difference was observed between LY367385 and Saline at any time points (4, 8 h, 1, 3, and 7 days). UL, unilateral labyrinthectomy; UBC, unipolar brush cells; DHPG, mGluR1α agonist; LY367385, mGluR1α antagonist; Control: UL + saline group; DHPG: UL + DHPG group; LY367385: UL + LY367385 group. ns, indicates no statistical difference; *p < 0.05; **p < 0.01; ****p < 0.0001.

To investigate the effect of mGluR1α agonist and antagonist, DHPG or LY367385 was injected into the flocculus 10 min before behavior analyses. Consistent with mGluR1α knockdown in the flocculus, control and LY367385‐injected groups showed VC over time (Figure 4; Two‐way ANOVA). Nonetheless, the recovery was significantly delayed in terms of nystagmus, postural asymmetry, and head tilt, especially 3 days after UL, but was comparable in terms of the result of tail‐hanging test. These findings suggested that mGluR1α inhibition might affect the chronic but not acute phase of VC. Interestingly, we found that ipsilateral administration of mGluR1α agonist DHPG into the flocculus significantly accelerated VC in both acute phase, as exhibited by all behavior analyses, Nevertheless, no significant difference was observed 3 days after UL in nystagmus and head tilt, and 7 days after UL in terms of postural asymmetry and tail hanging test results, as compared with the control group. These findings might be ascribed to the ceiling effect of VC. These results suggest that the activation of mGluR1α in the flocculus might be a feasible strategy for promoting VC.

### 3.5 Effects of different pharmacological interventions on the levels of PI/p‐ERK1/2 at different time points after UL

**FIGURE 5:**
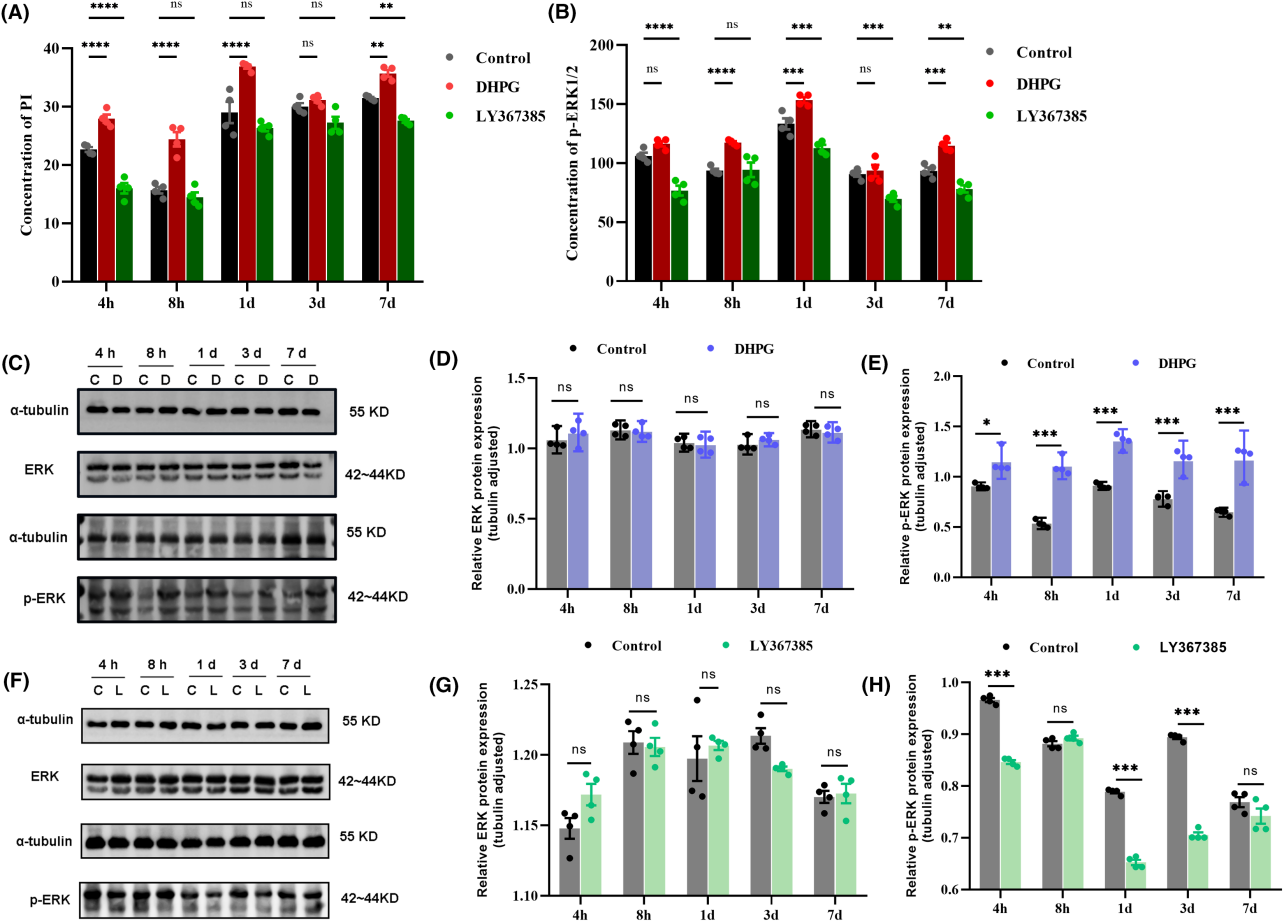
Effects of drug stimulations on PI/p‐ERK1/2 levels at various time points post‐UL. (A) DHPG increased while LY367385 decreased the PI level in the cerebellar flocculus of rats, compared to the UL + Saline group. Two‐way ANOVA showed a significant effect of drug stimulation (*F* (8,45) = 5.838, *p* < 0.0001). Bonferroni post‐hoc tests showed significant differences between the UL + Saline and UL + DHPG groups at 4, 8 h, 1, and 7 days (4 h, *p* < 0.0001; 8 h, *p* < 0.0001; 1 day, *p* < 0.0001; 7 days, *p* = 0.0019), but no significant difference at 3 days after UL (3 days, *p* = 0.9808). There were significant differences between the UL + Saline and UL + LY367385 groups at 4 h and 7 days (4 h, *p* < 0.0001; 7 days, *p* = 0.0041), but no significant difference at 8 h, 1, or 3 days after UL (8 h, *p* = 0.8851; 1 day, *p* = 0.0741; 3 days, *p* = 0.0629). (B) Compared to the Saline group, the p‐ERK1/2 level was increased in the cerebellar flocculus of rats in the DHPG group, while it was decreased in the LY367385 group. Two‐way ANOVA indicated a significant effect of DHPG drug stimulation (*F* (8,45) = 4.166, *p* = 0.0009). Bonferroni post‐hoc analysis showed significant differences between the UL + Saline group and the UL + DHPG group at 8 h, 1, and 7 days (8 h, *p* < 0.0001; 1 day, *p* = 0.000; 7 days, *p* = 0.0001), but no significant differences at 4 h and 3 days after UL (4 h, *p* = 0.0858; 3 days, *p* = 0.7964). Significant differences were observed between the UL + Saline group and the UL + LY367385 group at 4 h, 1, 3, and 7 days after UL (4 h, *p* < 0.0001; 1 day, *p* = 0.0002; 3 days, *p* = 0.0001, 7 days, *p* < 0.0005), but not at 8 h after UL (8 h, *p* = 0.9917). (C) The protein levels of ERK and p‐ERK in the cerebellar flocculus of rats, ERK were no change and p‐ERK were increased in DHPG‐injected Rat. (D) The quantification of ERK levels (*n* = 4 rat/group, two‐way ANOVA, *F* (4, 30) = 0.8065, *p* = 0.538). (E) The quantification of p‐ERK levels (*n* = 4 rat/group, two‐way ANOVA, *F* (4, 30) = 4.647, *p* = 0.005). (F) The protein levels of ERK and p‐ERK in the cerebellar flocculus of rats, ERK showed no change and p‐ERK was increased in LY367385‐injected Rat. (G) The quantification of ERK levels (*n* = 4 rat/group, two‐way ANOVA, *F* (4, 30) = 2.579, *p* = 0.058). (H) The quantification of p‐ERK levels (*n* = 4 rat/group, two‐way ANOVA, *F* (4, 30) = 74.300, *p* < 0.0001). UL, unilateral labyrinthectomy; UBC, unipolar brush cells; DHPG, mGluR1α agonist; LY367385, mGluR1α antagonist; Control: UL+ Saline group; DHPG: UL+ DHPG group; LY367385: UL+ LY367385 group; C: control group; D: DHPG group; L: LY367385 group. ns, indicates no statistical difference, **p < 0.01, ***p < 0.001, ****p < 0.0001.

The effects of DHPG and LY367385 on downstream signaling molecules PI and p‐ERK1/2 were measured using enzyme‐linked immunosorbent assay (ELISA) at different time points after UL. Changes in mGluR1α receptor signaling in the cerebellar flocculus were found to be correlated with VC‐related behavioral alternation. DHPG increased the PI and p‐ERK1/2 levels mediated by mGluR1α in the cerebellar flocculus of rats, while LY367385 decreased their levels, as compared to the UL+ Saline group (Figure 5A,B). In addition, we performed western blot analysis to verify the protein levels of both p‐ERK and total ERK (Figure 5C–H). The western blot results confirmed our observations as presented in Figure 5. These results suggested that the downstream pathway of mGluR1α, and PI/p‐ERK1/2 might mediate the effect of mGluR1α on VC in the flocculus.

## 4 DISCUSSION

This study demonstrated that mGluR1α plays a role in the cerebellar UBCs and exerts an impact on VC. By using a knockdown model and relevant pharmacological interventions, the study investigated the involvement of the mGluR1/IP3/ERK signaling pathway in the process of VC. The results unequivocally showed that mGluR1α activity played a critical part in ON UBCs in the mediation of VC. Activated mGluR1α increased ON UBC activity and phosphorylation of ERK, while inhibition led to a decrease in their activity. These findings provided an insight into the underlying mechanisms of VC and highlighted the significance of mGluR1α signaling in the pathogenesis of VC. Notably, the study employed a rigorous experimental design, and the results paved the way for future investigations about the therapeutic strategies targeting the mGluR1α pathway for promoting recovery following vestibular damage.

Firstly, we employed shRNA targeting sequences to selectively knock down the mGluR1α gene specifically harbored by ON UBCs, which serve as a dynamic network for signal transmission between granule cells and mossy fibers. By injecting AAV virus into the cerebellar flocculus of rats to induce a down‐regulated mGluR1α expression, we upset the potential balance between bilateral MVN neurons. This knockdown interfered with the inhibitory effect of the flocculus, leading to a profound postural imbalance. ON UBCs, which express mGluR1α, receive signals from diverse/multiple regions of the central nervous system through mossy fibers and relay them to granule cells.^8^ Granular cells transmit signals to Purkinje cells via parallel fibers. Of note, Purkinje cells, serving as the exclusive output neurons in the cerebellar cortex, exert an inhibitory control over the MVN in the vestibular cerebellar pathway. This intricate signaling cascade ultimately impacts the regulation of plasticity of the neurons in the vestibular cerebellar pathway.

Secondly, pharmacological interventions were used to examine the mechanism of VC. DHPG increased the activity of ON UBCs, while LY367385 reduced their activity, as evidenced by a change in the number of mGluR1α and c‐fos co‐localized positive neurons. C‐fos was successfully used as a marker of neuronal activation in a wide range of studies.^18^ In addition, UL induces c‐fos expression, which is a marker for the transmission of vestibular pathway signals.^19^, ^20^ Notably, c‐fos has been widely utilized to identify activated vestibular central neurons in the cases of unilateral or bilateral damage or inactivation of receptor hair cells, individual terminal organs, the entire labyrinth, or vestibular nerves. Its expression has been shown to play a pivotal role in VC.^20^, ^21^, ^22^ Previous investigations have revealed the expression of c‐fos in the vestibular‐nucleus‐olivocerebellar pathway following unilateral vestibular damage, suggesting the involvement of cerebellar pathways in the initial stage of VC.^23^, ^24^ During the acute phase, c‐fos and p‐ERK exhibited asymmetrical expression in the two sides of the MVN area. However, this asymmetry diminished 1 week later.^25^, ^26^, ^27^, ^28^

Furthermore, we observed that the mGluR1α agonist, DHPG, exerted a beneficial effects on VC‐related behavioral phenotypes, whereas the antagonist LY367385 delayed the recovery of these phenotypes. These findings further supported the involvement of the mGluR1/IP3/ERK signaling pathway in VC. Inoue et al.,^29^ by using microanalysis, found that glutamate concentration was decreased in the injured side of the MVN within 4 h after UL, and gradually returned to normal levels after 12 h. The difference in glutamate concentration between the two sides of the MVN was correlated with changes in spontaneous nystagmus. Other biochemical alterations might be associated with the glutamate release and its impact on VC, including the activation/inhibition of second messengers, protein kinases, and phosphorylation. Notably, a study showed that, in P8 mouse pups, mGluR1 signaling played a critical role in the growth and survival of Purkinje cells, particularly during early developmental stages.^30^ Another study demonstrated that, during later developmental stages, mGluR1 signaling regulated dendritic expansion in small cerebellar slice cultures and treatment with DHPG to induce mGluR1 signaling pathway resulted in a significant reduction in Purkinje cell dendritic growth.^31^

**FIGURE 6:**
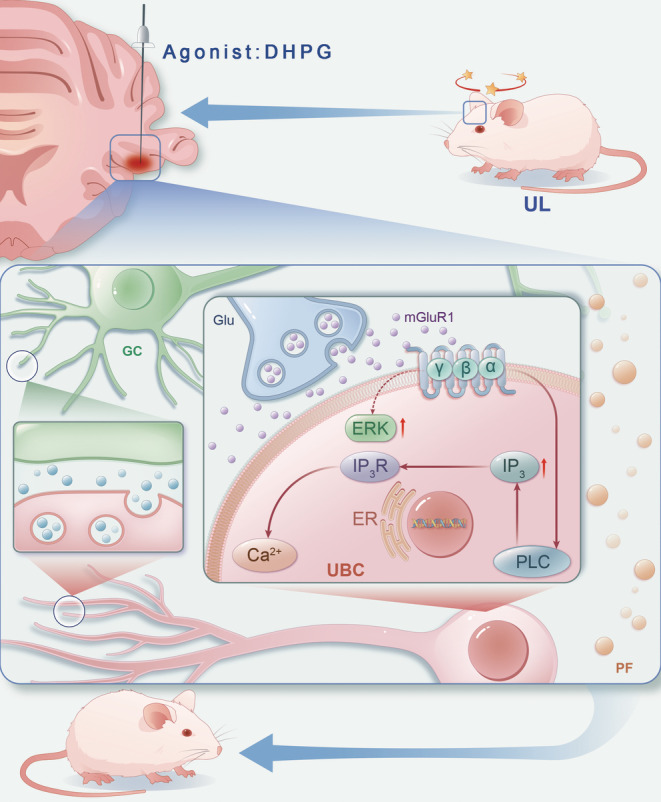
Algorithm of the mGluR1/IP3/ERK signaling pathway in cerebellar ON UBCs. Mossy fibers transmit signals to UBCs, which in turn relay them to granule cells or other UBCs and ultimately to Purkinje cells via parallel fibers. Purkinje cells, as the sole output neurons in the cerebellar cortex, inhibit the MVN in the vestibular‐cerebellar pathway. ON UBCs exhibit prolonged depolarization response to mossy fiber input, primarily mediated by mGluR1α receptors. Activation of mGluR1α triggers intracellular signaling through G proteins, PKC, IP3, and ERK, resulting in increased calcium efflux. The mGluR1α activity in cerebellar ON UBCs may crucial for mediating VC via the mGluR1/IP3/ERK pathway. UL, unilateral labyrinthectomy; UBC, unipolar brush cells; DHPG, mGluR1α agonist; MVN, medial vestibular nuclei; PKC, protein kinase C; ERK, extracellular signal‐regulated kinase; IP3, inositol 1,4,5‐triphosphate; IP3R, inositol 1,4,5‐triphosphate receptor, GC, granular cell; Glu, glutamic acid; PF, parallel fibers; ER, endoplasmic reticulum; VC, vestibular compensation.

In addition, our study indicated that VC might be associated with alterations in the phosphoinositide (PI) and p‐ERK1/2 pathways mediated by mGluR1α. Teleuca et al.^32^ utilized in vivo techniques to evaluate PI levels and investigated potential impairments in spatial learning and memory. Their primary objective was to examine the influence of changes in mGluR1 signaling on the PI pathway, as well as the MAP kinase and phosphatidylinositol‐3‐kinase pathways, in the hippocampus and prefrontal cortex. Glutamate, a major excitatory neurotransmitter, is essential for transmission of excitatory input signals from peripheral vestibular receptors to central vestibular neurons and for excitatory synaptic inputs in vestibular commissural fibers.^33^, ^34^ Kolber et al.^35^ demonstrated that, under baseline conditions, mGluR1 expression in the right amygdala exceeded that in the left, suggesting the lateralization of amygdala function was involved in pain processing. They also confirmed the mGluR1/ERK1/2 plays a role in the mediation of pain processing in the central nucleus of the amygdala. ERKs play a crucial role in transducing glutamate signals to the nucleus by phosphorylating nuclear transcription factors and mediating the transcription of c‐fos. Glutamate‐induced elevation of intracellular Ca^2+^ concentration in neurons activates the Ras/ERK signaling pathway.^36^ Based on our findings, mGluR1α mediates prolonged depolarization in ON UBCs, activating G proteins, PKC, IP3, ERK, leading to increased calcium efflux, crucial for VC, we proposed that ON UBCs may positively influence the cerebellar flocculus during VC through the mGluR1/IP3/ERK signaling pathway (Figure 6).

## 5 CONCLUSION

This study aimed to elucidate the role and underlying mechanism of mGluR1α in VC, further revealing that the mGluR1/IP3/ERK signaling pathway mediated the effects of mGluR1α on VC. Collectively, these findings, from a novel perspective, provided further insight into the molecular mechanisms underlying VC, and potential therapeutic targets for promoting recovery from peripheral vestibular damage.

## AUTHOR CONTRIBUTIONS

Conceptualization, S.Z. and D.L.; methodology, D.L.; software, D.L. and J.W.; validation, D.L. and J.W.; formal analysis, E.T.; writing–original draft preparation, D.L.; writing–review and editing, D.L. and J.W.; supervision, J.C.; project administration, Y.L. and W.K.; funding acquisition, S.Z. All authors have read and agreed to the published version of the manuscript.

## CONFLICT OF INTEREST STATEMENT

The authors declare no conflict of interest.

## Supporting information


Data S1

## Data Availability

Not applicable.

## References

[1] Arenz A , Silver RA , Schaefer AT , Margrie TW . The contribution of single synapses to sensory representation in vivo. Science. 2008;321(5891):977‐980. 18703744 10.1126/science.1158391 PMC2771362

[2] Barmack NH , Baughman RW , Errico P , Shojaku H . Vestibular primary afferent projection to the cerebellum of the rabbit. J Comp Neurol. 1993;327(4):521‐534. 7680050 10.1002/cne.903270405

[3] Barmack NH , Baughman RW , Eckenstein FP , Shojaku H . Secondary vestibular cholinergic projection to the cerebellum of rabbit and rat as revealed by choline acetyltransferase immunohistochemistry, retrograde and orthograde tracers. J Comp Neurol. 1992;317(3):250‐270. 1577999 10.1002/cne.903170304

[4] Borges‐Merjane C , Trussell LO . ON and OFF unipolar brush cells transform multisensory inputs to the auditory system. Neuron. 2015;85(5):1029‐1042. 25741727 10.1016/j.neuron.2015.02.009 PMC4370778

[5] Russo MJ , Yau HJ , Nunzi MG , Mugnaini E , Martina M . Dynamic metabotropic control of intrinsic firing in cerebellar unipolar brush cells. J Neurophysiol. 2008;100(6):3351‐3360. 18945818 10.1152/jn.90533.2008 PMC2604862

[6] van Dorp S , de Zeeuw CI . Variable timing of synaptic transmission in cerebellar unipolar brush cells. Proc Natl Acad Sci USA. 2014;111(14):5403‐5408. 24706875 10.1073/pnas.1314219111 PMC3986201

[7] Zampini V , Liu JK , Diana MA , Maldonado PP , Brunel N , Dieudonné S . Mechanisms and functional roles of glutamatergic synapse diversity in a cerebellar circuit. Elife. 2016;5:5. 10.7554/eLife.15872 PMC5074806 27642013

[8] Liu D , Wang J , Zhou L , et al. Differential modulation of cerebellar flocculus unipolar brush cells during vestibular compensation. Biomedicine. 2023;11(5):1298. 10.3390/biomedicines11051298 PMC10215535 37238967

[9] Gliddon CM , Sansom AJ , Smith PF , Darlington CL . Effects of intra‐vestibular nucleus injection of the group I metabotropic glutamate receptor antagonist AIDA on vestibular compensation in Guinea pigs. Exp Brain Res. 2000;134(1):74‐80. 11026728 10.1007/s002210000437

[10] Cartmell J , Schoepp DD . Regulation of neurotransmitter release by metabotropic glutamate receptors. J Neurochem. 2000;75(3):889‐907. 10936169 10.1046/j.1471-4159.2000.0750889.x

[11] Nicoletti F , Bockaert J , Collingridge GL , et al. Metabotropic glutamate receptors: from the workbench to the bedside. Neuropharmacology. 2011;60(7–8):1017‐1041. 21036182 10.1016/j.neuropharm.2010.10.022 PMC3787883

[12] C. National Research Council Committee for the update of the guide for the, A. use of laboratory. The National Academies Collection: reports funded by National Institutes of Health [M] . Guide for the Care and Use of Laboratory Animals. National Academies Press; 2011.

[13] Zhou W , Zhou LQ , Shi H , et al. Expression of glycine receptors and gephyrin in rat medial vestibular nuclei and flocculi following unilateral labyrinthectomy. Int J Mol Med. 2016;38(5):1481‐1489. 28026001 10.3892/ijmm.2016.2753 PMC5065303

[14] Zhou L , Zhou W , Zhang S , et al. BDNF signaling in the rat cerebello‐vestibular pathway during vestibular compensation: BDNF signaling in vestibular compensation. FEBS J. 2015;282(18):3579‐3591. 26111610 10.1111/febs.13360

[15] Lindner M , Gosewisch A , Eilles E , et al. Ginkgo biloba extract EGb 761 improves vestibular compensation and modulates cerebral vestibular networks in the rat. Front Neurol. 2019;10:147. 30858822 10.3389/fneur.2019.00147 PMC6397839

[16] Chen ZP , Zhang XY , Peng SY , et al. Histamine H1 receptor contributes to vestibular compensation. J Neurosci. 2019;39(3):420‐433. 30413645 10.1523/JNEUROSCI.1350-18.2018 PMC6335742

[17] Rastoldo G , Marouane E , El‐Mahmoudi N , et al. L‐thyroxine improves vestibular compensation in a rat model of acute peripheral vestibulopathy: cellular and behavioral aspects. Cell. 2022;11(4):684. 10.3390/cells11040684 PMC8869897 35203333

[18] Herdegen T , Leah JD . Inducible and constitutive transcription factors in the mammalian nervous system: control of gene expression by Jun, Fos and Krox, and CREB/ATF proteins. Brain Res Brain Res Rev. 1998;28(3):370‐490. 9858769 10.1016/s0165-0173(98)00018-6

[19] Kaufman GD , Anderson JH , Beitz AJ . Fos‐defined activity in rat brainstem following centripetal acceleration. J Neurosci. 1992;12(11):4489‐4500. 1432106 10.1523/JNEUROSCI.12-11-04489.1992 PMC6576004

[20] Holstein GR , Friedrich VL Jr , Martinelli GP , et al. Fos expression in neurons of the rat vestibulo‐autonomic pathway activated by sinusoidal galvanic vestibular stimulation. Front Neurol. 2012;3:4. 22403566 10.3389/fneur.2012.00004 PMC3289126

[21] Shinder ME , Perachio AA , Kaufman GD . VOR and Fos response during acute vestibular compensation in the Mongolian gerbil in darkness and in light. Brain Res. 2005;1038(2):183‐197. 15757634 10.1016/j.brainres.2005.01.043

[22] Kim MS , Kim JH , Jin YZ , Kry D , Park BR . Temporal changes of cFos‐like protein expression in medial vestibular nuclei following arsanilate‐induced unilateral labyrinthectomy in rats. Neurosci Lett. 2002;319(1):9‐12. 11814641 10.1016/s0304-3940(01)02422-3

[23] Jin YZ , Jin GS , Kim MS , et al. Role of Central Vestibular Pathway on Control of Blood Pressure During Acute Hypotension in Rats. 2005.

[24] Kim MS , Hyo Kim J , Kry D , et al. Effects of acute hypotension on expression of cFos‐like protein in the vestibular nuclei of rats. Brain Res. 2003;962(1–2):111‐121. 12543461 10.1016/s0006-8993(02)03977-x

[25] Kim MS , Jin BK , Chun SW , et al. Effect of MK801 on cFos‐like protein expression in the medial vestibular nucleus at early stage of vestibular compensation in uvulonodullectomized rats. Neurosci Lett. 1997;231(3):147‐150. 9300643 10.1016/s0304-3940(97)00550-8

[26] Li XL , Nian B , Jin Y , et al. Mechanism of glutamate receptor for excitation of medial vestibular nucleus induced by acute hypotension. Brain Res. 2012;1443:27‐33. 22305141 10.1016/j.brainres.2012.01.020

[27] Kitahara T , Takeda N , Saika T , et al. Role of the flocculus in the development of vestibular compensation: immunohistochemical studies with retrograde tracing and flocculectomy using Fos expression as a marker in the rat brainstem. Neuroscience. 1997;76(2):571‐580. 9015339 10.1016/s0306-4522(96)00374-0

[28] Li YX , Hashimoto T , Tokuyama W , Miyashita Y , Okuno H . Spatiotemporal dynamics of brain‐derived neurotrophic factor mRNA induction in the vestibulo‐olivary network during vestibular compensation. J Neurosci. 2001;21(8):2738‐2748. 11306626 10.1523/JNEUROSCI.21-08-02738.2001 PMC6762513

[29] Inoue S , Yamanaka T , Kita T , Nakashima T , Hosoi H . Glutamate release in the rat medial vestibular nucleus following unilateral labyrinthectomy using in vivo microdialysis. Brain Res. 2003;991(1–2):78‐83. 14575879 10.1016/j.brainres.2003.08.002

[30] Adcock KH , Metzger F , Kapfhammer JP . Purkinje cell dendritic tree development in the absence of excitatory neurotransmission and of brain‐derived neurotrophic factor in organotypic slice cultures. Neuroscience. 2004;127(1):137‐145. 15219676 10.1016/j.neuroscience.2004.04.032

[31] Sirzen‐Zelenskaya A , Zeyse J , Kapfhammer JP . Activation of class I metabotropic glutamate receptors limits dendritic growth of Purkinje cells in organotypic slice cultures. Eur J Neurosci. 2006;24(11):2978‐2986. 17156359 10.1111/j.1460-9568.2006.05196.x

[32] Teleuca AE , Alemà GS , Casolini P , et al. Changes in mGlu5 receptor signaling are associated with associative learning and memory extinction in mice. Life. 2022;12(3):463. 35330215 10.3390/life12030463 PMC8955168

[33] Choi MA , Lee JH , Hwang JH , Choi SJ , Kim MS , Park BR . Signaling pathway of glutamate in the vestibular nuclei following acute hypotension in rats. Brain Res. 2008;1229:111‐117. 18639534 10.1016/j.brainres.2008.06.088

[34] Smith PF . Why the cerebellar shutdown/clampdown hypothesis of vestibular compensation is inconsistent with neurophysiological evidence. J Vestibular Res. 2020;30(5):295‐303. 10.3233/VES-200715 33044204

[35] Kolber BJ , Montana MC , Carrasquillo Y , et al. Activation of metabotropic glutamate receptor 5 in the amygdala modulates pain‐like behavior. J Neurosci. 2010;30(24):8203‐8213. 20554871 10.1523/JNEUROSCI.1216-10.2010 PMC2898903

[36] Vanhoutte P , Barnier JV , Guibert B , et al. Glutamate induces phosphorylation of Elk‐1 and CREB, along with c‐fos activation, via an extracellular signal‐regulated kinase‐dependent pathway in brain slices. Mol Cell Biol. 1999;19(1):136‐146. 9858538 10.1128/mcb.19.1.136 PMC83872

