# Supplementary material for: mGluR1/IP3/ERK signaling pathway regulates vestibular compensation in ON UBCs of the cerebellar flocculus
Source: CNS Neurosci Ther. 2023 Aug 25;30(2):e14419. doi: 10.1111/cns.14419 (PMC10848063; doi:10.1111/cns.14419)
Supplement: Supplementary file 1 — Data S1 [file CNS-30-e14419-s001.pdf]

Full unedited gel/blot for Figure 1G

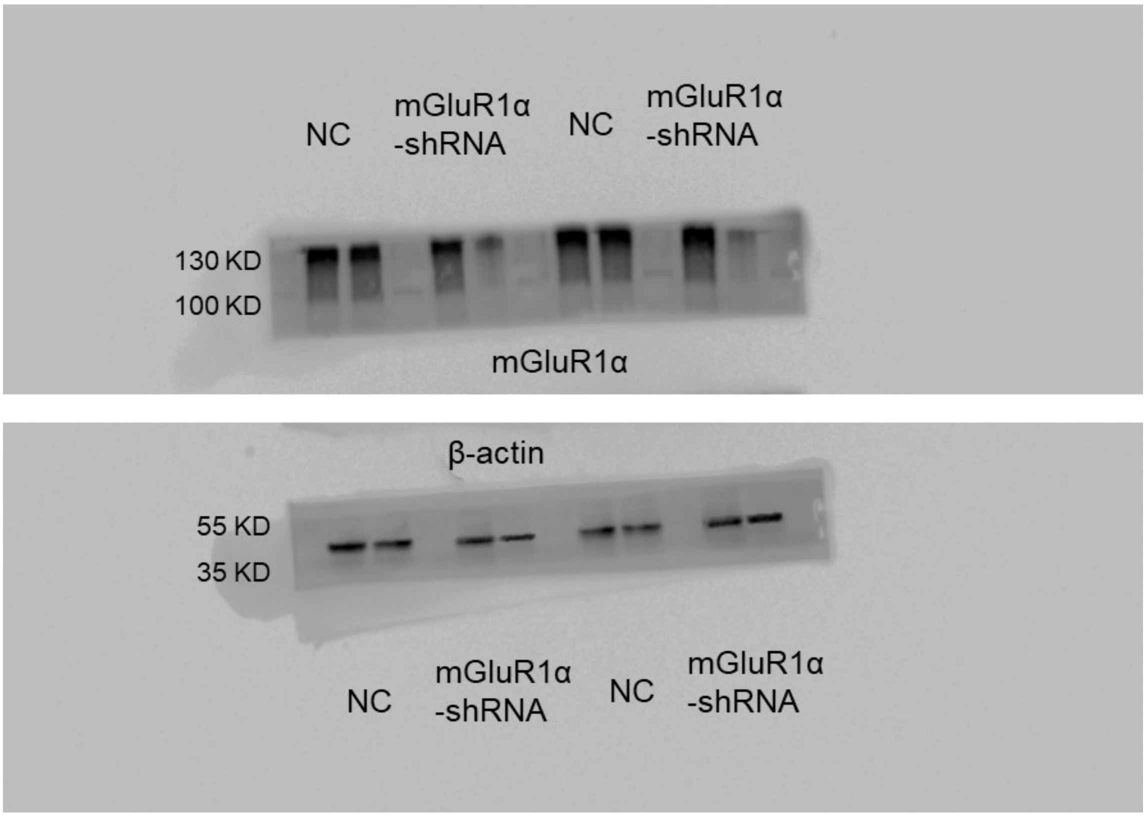

Full unedited gel/blot for Figure 5C

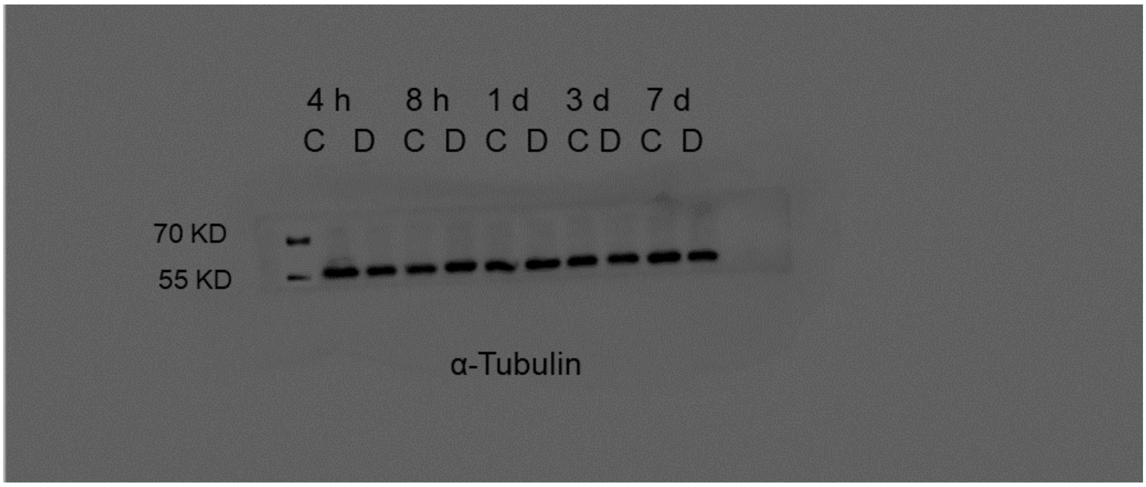

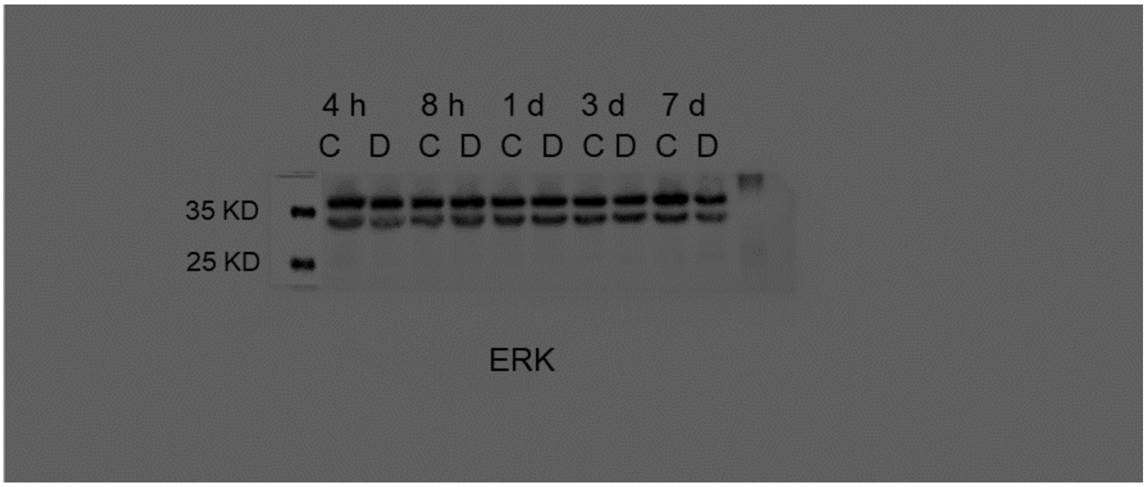
4 h 8 h 1 d 3 d 7 d
C D C D C D C D C D
35 KD
25 KD
ERK

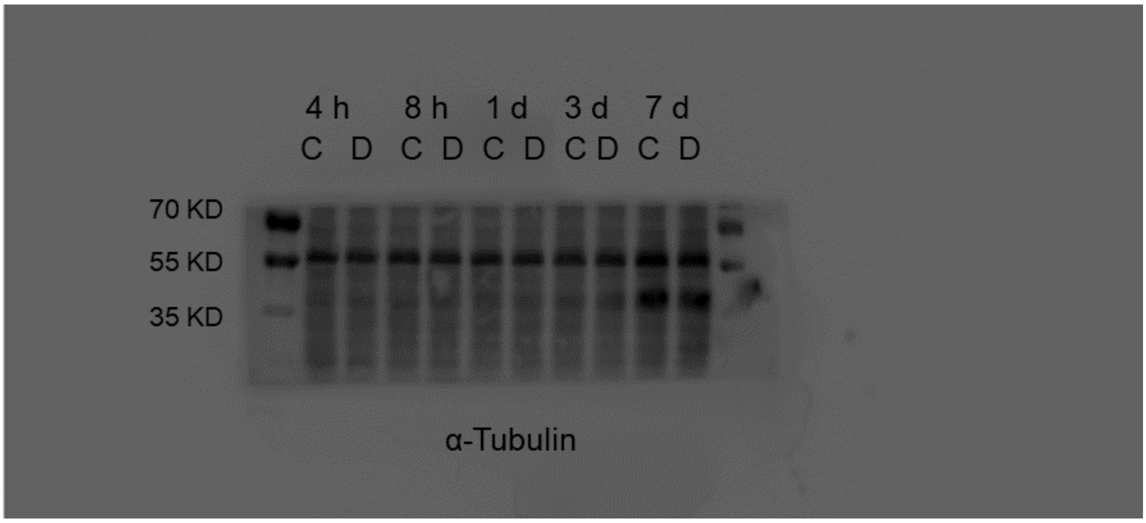
4 h 8 h 1 d 3 d 7 d
C D C D C D C D C D
70 KD
55 KD
35 KD
α-Tubulin

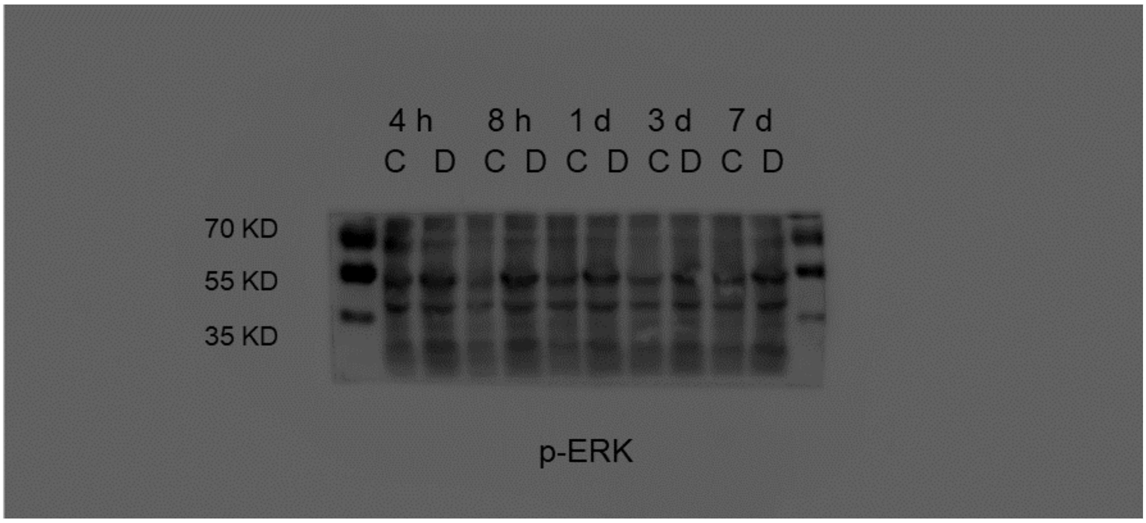
4 h 8 h 1 d 3 d 7 d
C D C D C D C D C D
70 KD
55 KD
35 KD
p-ERK

Full unedited gel/blot for Figure 5F

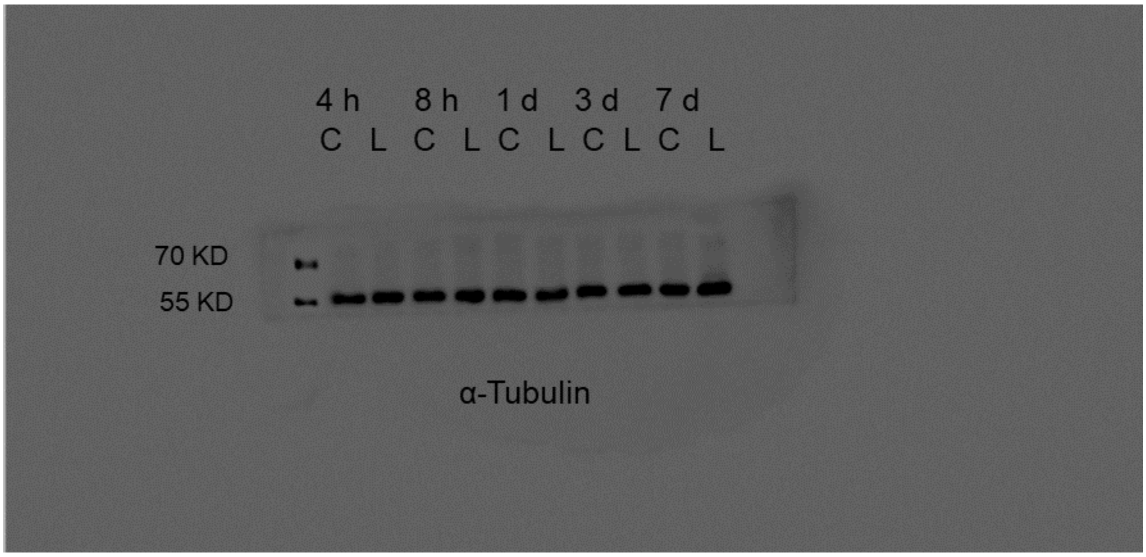

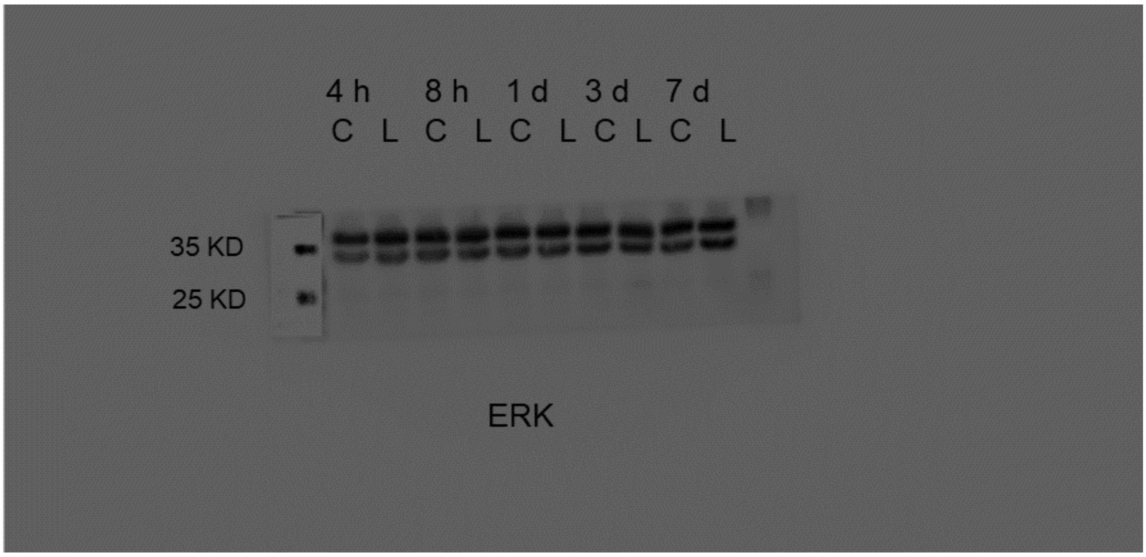

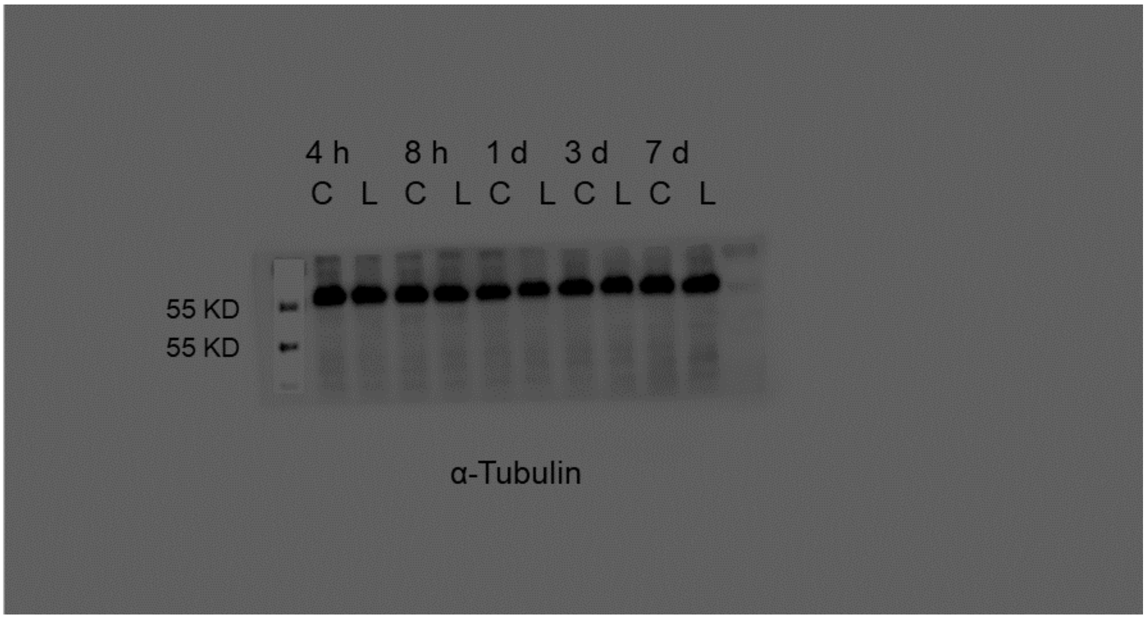
4 h 8 h 1 d 3 d 7 d
C L C L C L C L C L
55 KD
55 KD
α-Tubulin

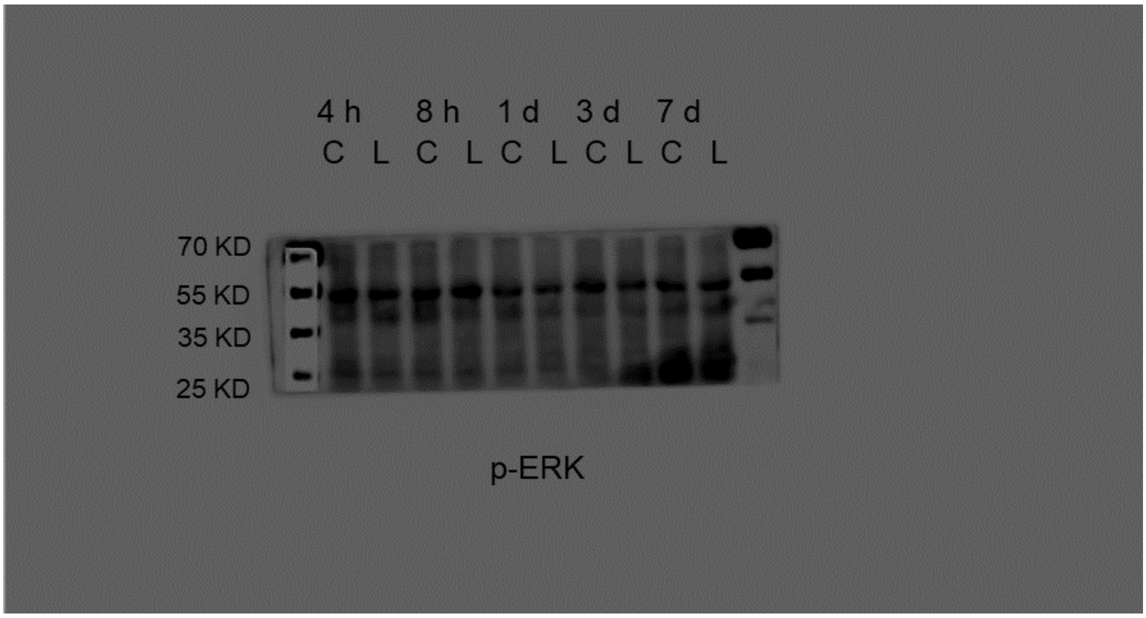
4 h 8 h 1 d 3 d 7 d
C L C L C L C L C L
70 KD
55 KD
35 KD
25 KD
p-ERK
